# Biofabrication of a Filtration Barrier by Integrating Electrospun Membranes and Flow in a Glomerular Co‐Culture

**DOI:** 10.1002/adhm.202501235

**Published:** 2025-07-03

**Authors:** Camilla Mussoni, Anna Rederer, Vladimir Stepanenko, Frank Würthner, Philipp Stahlhut, Jürgen Groll, Mario Schiffer, Taufiq Ahmad, Janina Müller‐Deile

**Affiliations:** ^1^ Department of Functional Materials in Medicine and Dentistry Institute of Functional Materials and Biofabrication (IFB) and Bavarian Polymer Institute (BPI) Julius‐Maximilians‐Universität Würzburg D‐97070 Würzburg Germany; ^2^ Department of Nephrology Medizinische Klinik 4 Uniklinikum Erlangen Friedrich‐Alexander‐Universität Erlangen‐Nürnberg 91054 Erlangen Germany; ^3^ Institut für Organische Chemie, Center for Nanosystems Chemistry (CNC) Julius‐Maximilians‐Universität Würzburg 97074 Würzburg Germany

**Keywords:** biofabrication, bioreactors, electrospinning, fenestrae, glomerular endothelial cells, glomerular filtration barrier, hiPSC‐derived podocytes

## Abstract

The glomerular filtration barrier (GFB), composed of glomerular endothelial cells, podocytes, and the glomerular basement membrane (GBM), is essential for selective filtration in the kidney. Damage to any GFB component results in proteinuria and kidney failure. To model glomerular pathophysiology and test therapies, this study introduces an ex vivo GFB model using electrospun poly‐L‐lactic acid fibers that mimic the GBM. The fibers are functionalized with polydopamine and gelatin, then seeded with human glomerular endothelial cells and podocytes on opposite sides. This biomimetic setup supports monolayer formation, cell‐type‐specific marker expression, and appropriate morphology of both cell types, including those derived from human induced pluripotent stem cells (hiPSCs). The model demonstrates bidirectional cell‐cell communication across the membrane. Permeability assays confirm size‐selective dextran filtration. Under flow conditions in a custom 3D‐printed micro‐bioreactor, endothelial cells develop fenestrae‐like structures, whereas podocytes form foot process‐like extension features, which are often lacking in static in vitro systems. This platform replicates critical features of the native GFB and provides a robust system for studying glomerular function, disease mechanisms, and therapeutic responses. Moreover, incorporating hiPSC‐derived podocytes enables exploration of patient‐specific mutations and personalized treatment strategies in kidney disease.

## Graphical abstract

An ex vivo glomerular filtration barrier is engineered using electrospun, biomimetic membranes seeded with human glomerular cells. The model supports fenestrated endothelium and podocyte foot processes under flow, mimics size‐selective filtration, and enables disease modeling with hiPSC‐derived podocytes, offering a powerful platform for studying kidney pathophysiology and personalized therapies.

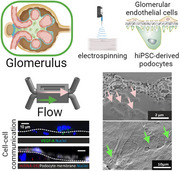

## 1 Introduction

Kidney glomeruli serve as the primary site for the selective ultrafiltration of blood, which is involved in the excretion of metabolic waste by allowing water and small molecules to pass while preventing the passage of larger proteins and blood cells.^[^ ^1^, ^2^ ^]^ The glomerular filtration barrier (GFB) is a tri‐layered structure composed of fenestrated endothelium, featuring 70–100 nm diameter fenestrations, a glomerular basement membrane (GBM) rich in extracellular matrix (ECM) proteins such as type IV collagen and laminin, and podocytes with interdigitating foot processes connected by slit diaphragms.^[^ ^1^, ^3^, ^4^, ^5^, ^6^ ^]^ This architecture ensures selectivity based on size and charge, enabling the daily filtration of ≈180 liters of primary urine from whole blood.^[^ ^1^, ^2^ ^]^ Any disruption in the components of the glomerulus could lead to proteinuria, ultimately resulting in kidney failure.^[^ ^7^ ^]^ Therefore, proper development, regulation, and cross‐communication among all layers of the GFB are essential for the maintenance and functionality of the barrier. Various glomerular diseases, such as diabetic nephropathy or primary glomerulonephropathies caused by autoimmune dysregulation or auto‐antibodies against components of the GFB, as well as genetic diseases due to mutations in podocyte genes or genes encoding proteins of the GBM, lead to impairments in GFB function, resulting in proteinuria and/or hematuria and progressive chronic kidney disease (CKD).  

There is a significant need for ex vivo models of the GFB to simulate physiological and pathophysiological conditions. However, conventional 2D culture methods are limited in their ability to replicate the complex physiology and microarchitectures of the GFB. Consequently, there is a growing demand for in vitro models that can accurately emulate both normal and pathological conditions. These models must retain essential features of the GFB, such as the fenestration of glomerular endothelial cells, the interdigitating foot processes of podocytes, and the capacity for size‐ and charge‐selective filtration. 

The advancement of GFB in vitro models has been fueled by the integration of 3D culture systems, organ‐on‐chip (OOC) platforms,^[^ ^8^, ^9^, ^10^, ^11^, ^12^, ^13^, ^14^, ^15^, ^16^, ^17^, ^18^ ^]^ and biomimetic materials like nanofibers and hydrogels,^[^ ^10^, ^18^, ^19^, ^20^, ^21^ ^]^ which enhance cell support, signaling, and morphogenesis. OOC platforms, capable of mimicking the intricate dynamic environment of the glomerulus, have emerged as transformative tools in preclinical research. Despite these developments, traditional OOC systems rely heavily on synthetic membranes such as polydimethylsiloxane (PDMS) and polycarbonate (PC), which are considerably thicker than human basement membranes, limiting physiological accuracy. These synthetic materials are non‐degradable, flat, and lack the essential topographical features needed to support proper ECM remodeling and transmembrane crosstalk, both crucial for studying tissue functions and disease mechanisms. Nanofiber scaffolds present a promising alternative to PDMS and PC membranes by providing ultrathin, biomimetic substrates with high surface area and adjustable porosity that more accurately replicate native tissue structures.^[^ ^22^ ^]^

Here, we developed a GFB using an electrospun poly‐L‐lactic acid (PLLA) membrane to mimic the GBM, which was seeded with human glomerular endothelial cells and human podocytes (either immortalized or hiPSC‐derived). Electrospun nanofibers provide numerous advantages over commercially available synthetic systems regarding size, shape, material variety, fiber morphology, orientations, and the modification of nanofibers with bioactive cues.^[^ ^22^ ^]^ By fabricating the artificial GBM in various orientations, we demonstrated that fiber topography impacts cell attachment and morphology. Nevertheless, we achieved highly viable cell monolayers exhibiting characteristic glomerular cell morphology and marker expression on the aligned membrane. The functional properties of the GFB model, such as barrier function, were assessed by measuring dextran passage through the filtration barrier. Additionally, we designed and fabricated a customized reusable bioreactor using 3D printing to apply flow conditions for three days. We observed fenestrae‐like pores organized in clusters on the surface of glomerular endothelial cells, confirming the maturation of cells within the system. Furthermore, we demonstrated glomerular cell‐cell communication within our model and selective permeability.

Thus, we present an in vitro model of the GFB that allows for flow conditions and measures both glomerular barrier function and permeability in healthy and diseased states. Using personalized hiPSC‐derived podocytes in this model enables individualized disease modeling in the future.

## 2 Results

### 2.1 Characterization of the Functionalized Electrospun Nanofibers

**Figure 1:**
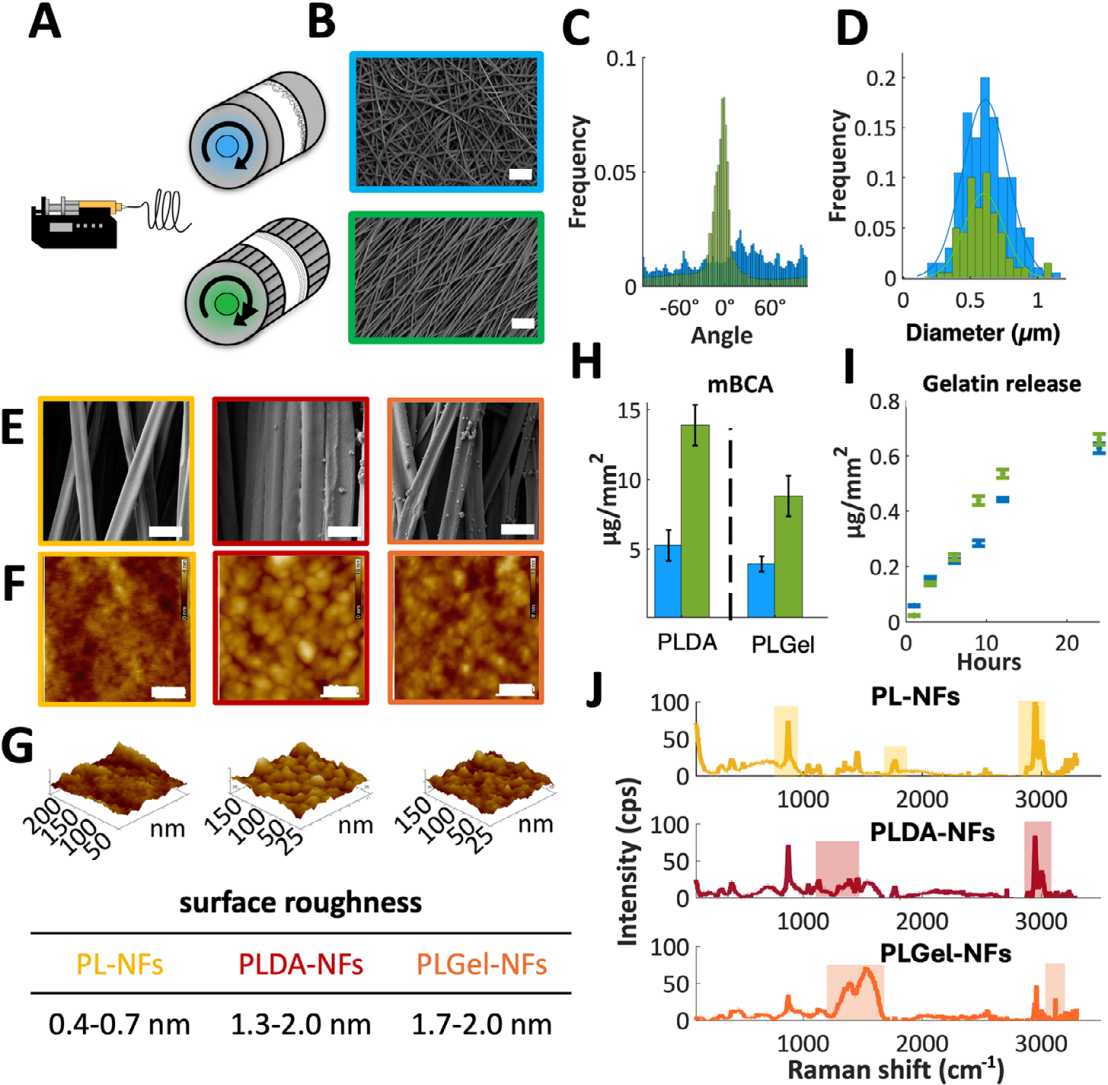
Characterization of nanofiber‐based artificial membrane. A) Schematic illustration of electrospinning random and aligned membranes using different mandrels. B) SEM images of random (blue) and aligned (green) nanofibers. Scale bar: 4 µm. C) Fiber alignment distribution for random (blue) and aligned (green) membranes. D) Fiber diameter distribution for random (blue) and aligned (green) mats. E) SEM images of PL‐NF, polydopamine PLDA‐NF, and gelatin PLGel‐NF coated fibers. Scale bar: 1 µm. F) AFM image of PL‐NF, polydopamine PLDA‐NF, and gelatin PLGel‐NF. Scale bar: 50 nm. G) AFM surface roughness analysis (Root Mean Square). H) mBCA quantification of coating for random (blue) and aligned (green) membranes. I) Gelatin release from the membrane over 24 h. J) Raman spectroscopy of surface chemistry for PL‐NF, PLDA‐NF, and PLGel‐NF. Abbreviations: AFM: atomic force microscope, PL‐NF: Poly‐L‐lactic acid nanofibers, PLDA‐NF: Polydopamine‐coated PLLA nanofibers, PLGel‐NF: Gelatin‐coated PLLA nanofibers, SEM: Scanning electron microscopy.

We utilized electrospinning to produce an artificial GBM, which was subsequently seeded with human glomerular endothelial cells and human immortalized or hiPSC‐derived podocytes on opposite sides of the membrane. Poly‐L‐Lactic Acid (PLLA) was chosen for electrospinning due to its enhanced mechanical properties, allowing us to achieve fibers in the nanometer to micrometer range while ensuring sufficient mechanical stability for handling. The polymer was electrospun in either a random or aligned topography, and cell behavior was analyzed to identify optimal conditions for our purposes (**Figure** **1**A,B). The electrospun mats exhibited an average thickness of 7 µm. For the aligned type, alignment is present in 86% of the fibers (Figure 1C). Both types of mats have an average fiber diameter of 0.6 µm (±0.4 µm for aligned, ±0.5 for random) (Figure 1D). The porosity of the mat was calculated to be ≈57% for random and 63% for aligned.

We modified the surface of PLLA nanofibrous membranes by immobilizing gelatin using polydopamine (PDA) chemistry. Scanning electron microscopy (SEM) imaging showed no significant changes in the diameter of the nanofibers before or after the coating (Figure 1E). Interestingly, atomic force microscopy (AFM) measurements indicated a change in surface roughness of the single fibers (root mean square), registering 0.4–0.7 nm for Poly‐L‐lactic acid nanofibers (PL‐NFs), 1.3–2.0 nm for polydopamine‐coated PLLA nanofibers (PLDA‐NFs), and 1.7–2.0 nm for gelatin‐immobilized PLLA nanofibers (PLGel‐NF) (Figure 1F,G). The quantification of the coating was performed using a micro‐BCA assay. This analysis demonstrated that 3.96 ± 0.55 µg mm⁻^2^ of gelatin is present on the random mats, and 8.82 ± 1.45 µg mm⁻^2^ on the aligned mats (Figure 1H). Measurement of gelatin release over time showed that up to 0.6 ± 0.02 µg mm⁻^2^ of gelatin detached from the mat within 24 h Figure 1I).

To demonstrate the technique's efficacy, we analyzed the fibers' surface using Raman spectroscopy. Characteristic Raman peaks were detected for the chemical bond molecules, confirming the properties of the surface chemistry (Figure 1J). PLLA typically peaks at 873 cm^−1^ C─COO, which can be attributed to the 1750 cm^−1^ identifies the C ═ O and at 2950 cm^−1^ to the CH_3_ chains. The dopamine spectrum differs for the presence of slightly higher peaks at 1500 and 1350 cm^−1^, suggestive of aliphatic and aromatic groups. Furthermore, the amine bond can be detected in the band of 3150 cm^−1^. The gelatin spectrum is mostly associated with the presence of ample peaks ≈1560, 1650, and 3100 cm^−1^ due to amide bonds.^[^ ^23^ ^]^

### 2.2 Human Glomerular Cells on the Artificial GBM

**Figure 2:**
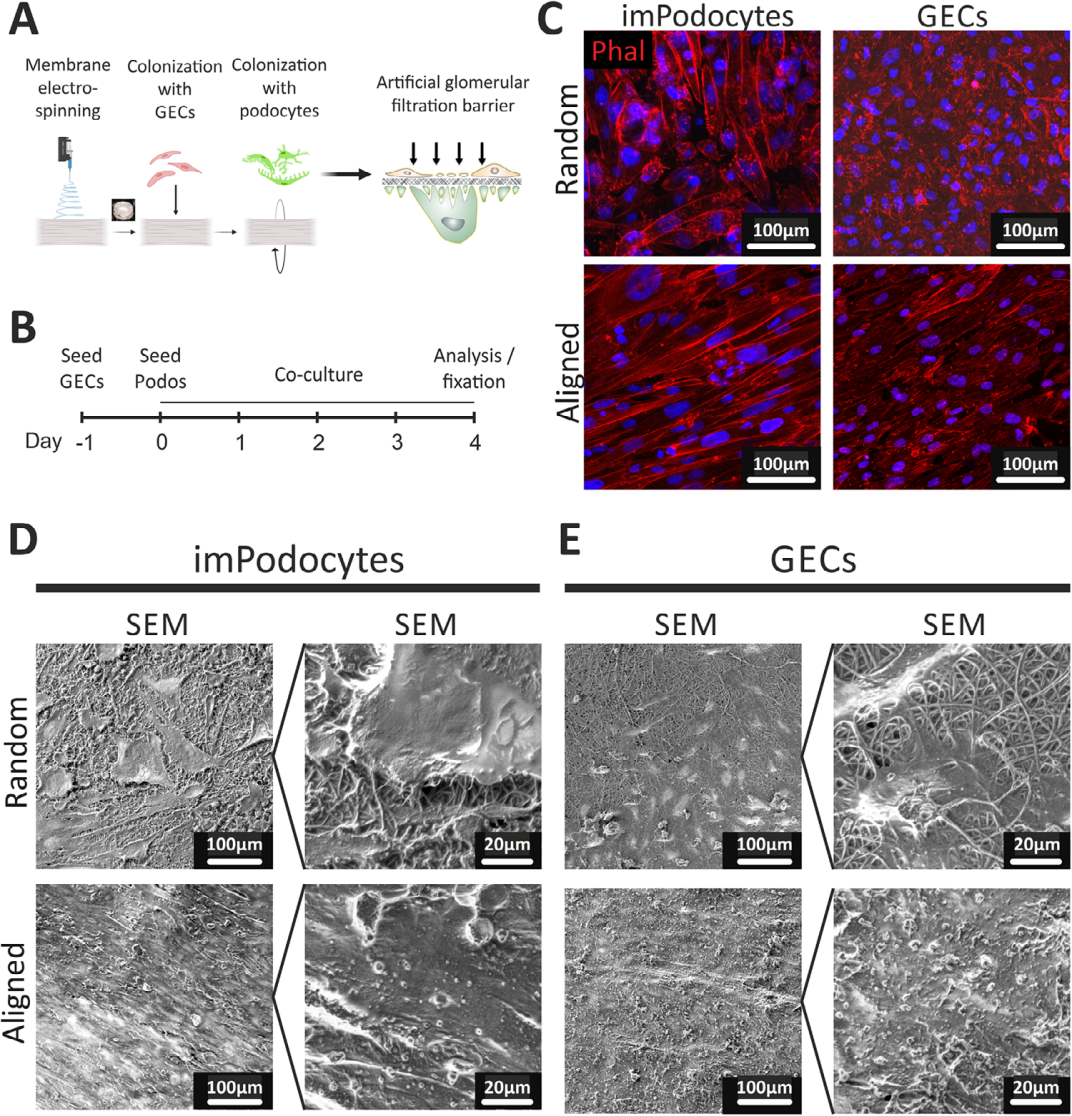
Co‐culture of human conditionally immortalized podocytes (imPodocytes) and glomerular endothelial cells (GECs) on the electrospun polydopamine‐ and gelatin‐coated membrane. A) Schematic protocol for membrane colonization with human GECs and imPodocytes from different sides of the membrane. Created in BioRender. Laptii, A. (2025) https://BioRender.com/g63h412. B) Schematic timeline of cell co‐culturing on the electrospun membrane. C,D) Cell morphology (C) and layer integrity of imPodocytes (D) and GECs E) on the membrane with random (upper panels) and aligned (bottom panels) topography visualized using confocal microscopy with phalloidin (Phal) staining (C) and SEM (D‐E). Scale bar 100 µm, 20 µm, respectively. E) Immunofluorescent staining for laminin 5 (Lam5; green), collagen IV (Col4; red), and DAPI nuclei staining (blue) of imPodocytes and GECs cultured on the membrane with random (top) or aligned (bottom) topography. Scale bar 100 µm.​ Abbreviations: GECs: glomerular endothelial cells, ImPodocytes: human conditionally immortalized podocytes, SEM: Scanning electron microscopy.

After these basic characterizations, the electrospun membrane was utilized for cell culture. To establish a tri‐layered model for the GFB with an electrospun PLLA membrane supporting the co‐culture of glomerular cells, the membrane was secured between two 3D‐printed supporting rings, allowing the seeding of glomerular endothelial cells and podocytes on opposite sides of the membrane (**Figure** **2**A,B). The fibers were either uncoated or functionalized with gelatin type B using polydopamine chemistry to enhance cell adhesion. By fabricating the artificial GBM with different topographies, we demonstrated that cell attachment and morphology were influenced by fiber topography and coating. Glomerular endothelial cells and immortalized podocytes cultured on a non‐coated membrane demonstrated insufficient formation of cell layers (Figure S1, Supporting Information).

We observed a high level of cell survival independently of fiber topography (indicated by live‐dead staining, Figure S2, Supporting Information). However, the immortalized podocyte cell layer showed considerable gaps between cells on a random membrane. Conversely, podocytes cultured on an aligned membrane exhibited a tight monolayer and elongated morphology, as shown by immunofluorescent staining and scanning electron microscopy (SEM) (Figure 2C,D). Likewise, glomerular endothelial cells formed a coherent monolayer only on the aligned membrane, not on the random membrane (Figure 2C,E).

Glomerular endothelial cells and podocytes are known to produce the extracellular matrix (ECM) of the GBM. We could show that important components of the mature GBM, such as laminin‐521 and collagen α3α4α5 (IV), were synthesized by our cells themselves within our model, especially when cells were cultured on aligned fibers (Figure S3, Supporting Information).

### 2.3 HiPSC‐Derived Podocytes Show Better Podocyte Characteristics Compared to imPodocytes

**Figure 3:**
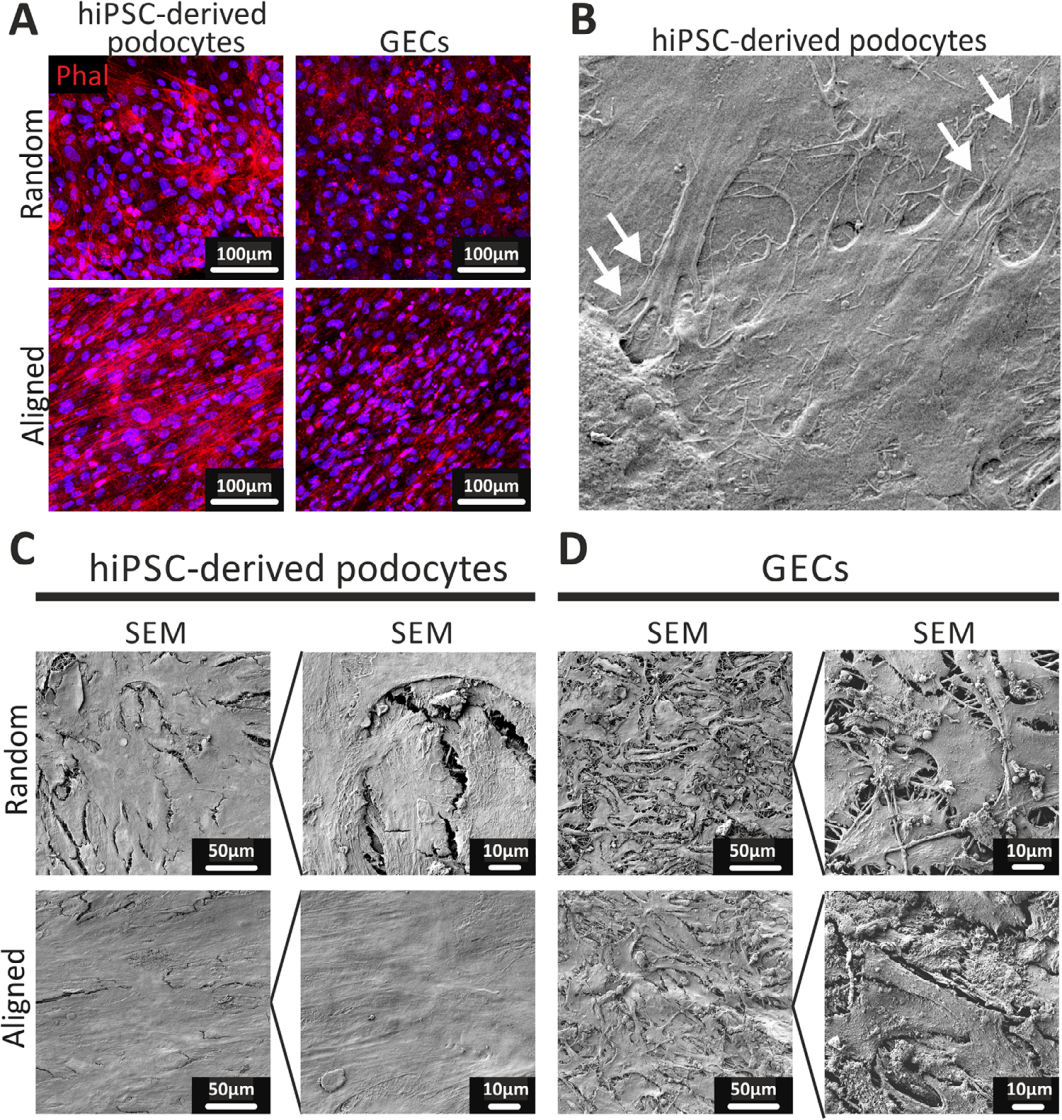
Co‐culture of hiPSC‐derived podocytes and GECs on the electrospun polydopamine‐ and gelatin‐coated membrane. A–D) Cell morphology (A) and layer integrity of hiPSC‐derived podocytes (C) and GECs (D) on the membrane with random (top) and aligned (bottom) topography visualized using confocal microscopy with Phal staining (A) and SEM (C‐D). (B): Podocyte‐like cell protrusions (white arrows) of the hiPSC‐derived podocytes co‐cultured with GECs on the membrane with aligned topography visualized using SEM.​ Abbreviations: GECs: glomerular endothelial cells, hiPSC: human induced pluripotent stem cells, SEM: Scanning electron microscopy.

Immortalized podocytes face several limitations: cells can dedifferentiate in culture, particularly upon reaching confluency, and various podocyte‐specific markers are either minimally expressed or entirely absent.^[^ ^24^, ^25^ ^]^ This raises concerns about the use of immortalized podocytes and their relevance for physiological, pathophysiological, and clinical research. Recently, we established a protocol for generating human podocytes‐ including patient‐specific podocytes‐ from a skin punch biopsy by episomal reprogramming of dermal fibroblasts into human‐induced pluripotent stem cells (hiPSCs), followed by differentiation into hiPSC‐derived podocytes (Figure S1A, Supporting Information).^[^ ^26^ ^]^ We introduced hiPSC‐derived podocytes into our in vitro model of the GFB. HiPSC‐derived podocytes demonstrated high detachment from the membrane when cultured as a monoculture (data not shown). In contrast, hiPSC‐derived podocytes cultured in combination with glomerular endothelial cells on the opposite side of the membrane displayed a high viability and formed a tight monolayer, at least for four days of culturing (**Figure** **3**A–C; Figure S2B, Supporting Information). Again, hiPSC‐derived podocytes were able to survive on both aligned and random fibers (Figure S2, Supporting Information), but culturing the cells on aligned fibers promoted higher cell confluency and improved monolayer formation of hiPSC‐derived podocytes (Figure 3C) and glomerular endothelial cells (Figure 3D). Indeed, podocytes generated by our protocol more closely resemble in vivo podocytes in terms of their morphological characteristics and the expression of podocyte‐specific markers.

**Figure 4:**
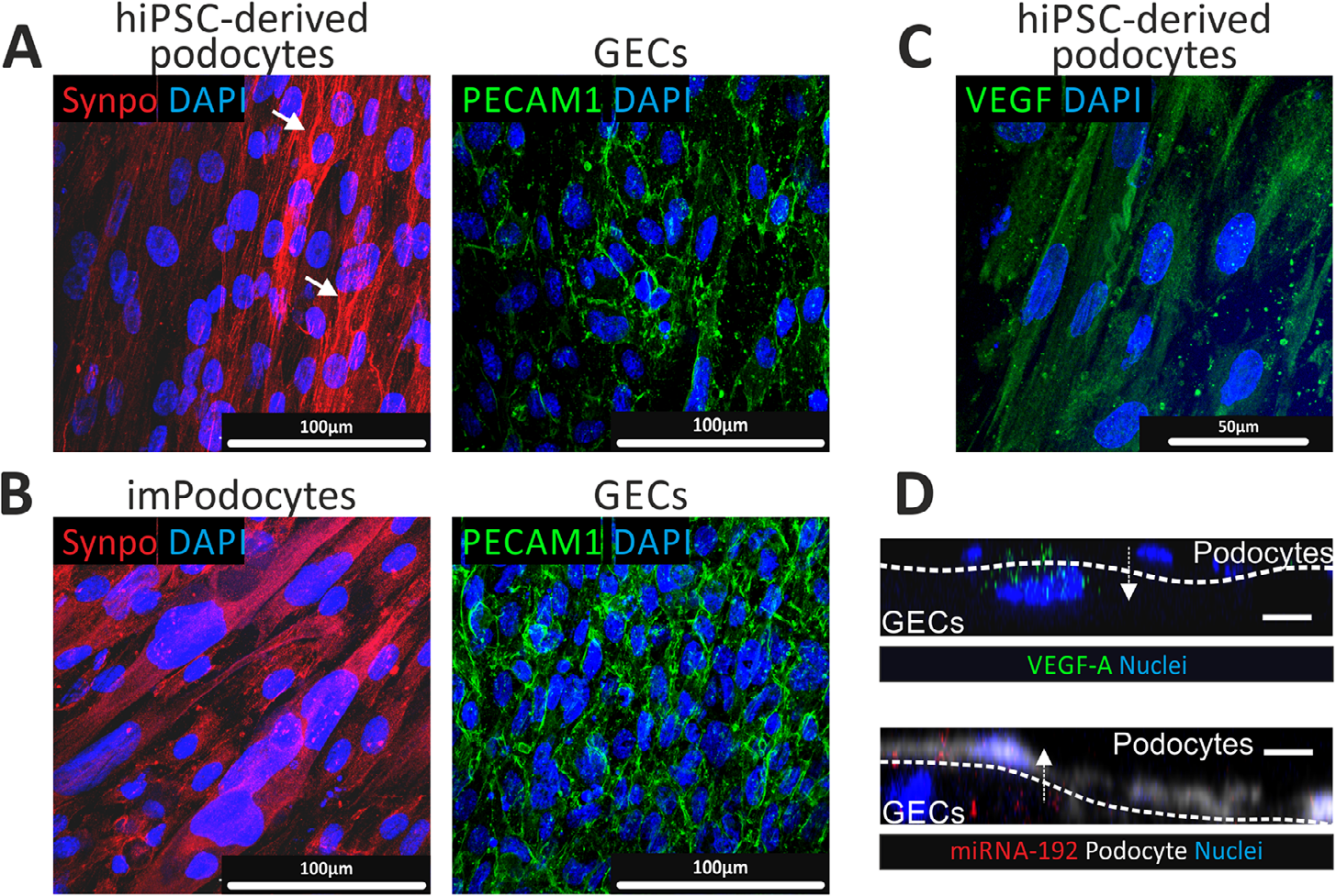
Maturation and intercellular communication of co‐cultured hiPSC‐derived podocytes or imPodocytes and GECs on the aligned membrane. A,B) Expression of the cell‐type specific markers in the co‐culture of hiPSC‐derived podocytes and GECs (A) as well as imPodocytes and GECs (B) cultured on the opposite sides of the electrospun polydopamine‐ and gelatin‐coated membrane with aligned topography. Membranes were stained against synaptopodin (Synpo, red) and PECAM1 (green) and visualized using confocal microscopy. ​ Abbreviations: GECs: glomerular endothelial cells, hiPSC: human induced pluripotent stem cells, ImPodocytes: human conditionally immortalized podocytes, PECAM1: Platelet and endothelial cell adhesion molecule 1.

The podocyte‐specific marker synaptopodin was expressed in a fibrous manner in hiPSC‐derived podocytes, while imPodocytes exhibited a diffuse expression of synaptopodin (**Figure** **4**A,B). The endothelial cell marker PECAM1 appeared as a zipper‐like structure along the edges of endothelial cells in both conditions (Figure 4A,B). Collagen IV was expressed by both cell types, whereas laminin 5 was secreted solely by hiPSC‐derived podocytes (Figure S3, Supporting Information). Furthermore, hiPSC‐derived podocytes displayed typical foot‐like processes between neighboring cells, which were not observed in immortalized podocytes (Figure 3B).

### 2.4 Cell Communication Through the Artificial GFB

Cell‐cell communication is crucial for the proper functioning of the GFB. Communication between glomerular endothelial cells and hiPSC‐derived podocytes occurs through the natural GBM.^[^ ^27^, ^28^ ^]^ It is well‐studied that glomerular endothelial cells require VEGF from podocytes for the formation of fenestrae. We confirmed that co‐cultured podocytes produced endogenous VEGF (Figure 4C). To track the transport of the podocyte‐produced VEGF, we electroporated podocytes with a plasmid encoding fluorescent VEGF‐A and co‐cultured the cells with glomerular endothelial cells on the membrane. Imaging the membrane from the endothelial side confirmed the uptake of podocyte‐derived VEGF‐A by glomerular endothelial cells (Figure 4D, top panel).

We also demonstrated cell‐to‐cell communication in the opposite direction. Glomerular endothelial cells and podocytes are known to communicate through microRNAs (miRs).^[^ ^29^ ^]^ We transfected glomerular endothelial cells with red fluorescent miR‐192 and co‐cultured these cells with podocytes on the artificial GBM. The uptake of the red fluorescent endothelial cell‐derived miR in podocytes was detected (Figure 4D, bottom panel).

Thus, we showed that cell‐to‐cell communication between glomerular endothelial cells and podocytes occurs in both directions.

### 2.5 Artificial GFB Shows Size‐Selective Permeability

**Figure 5:**
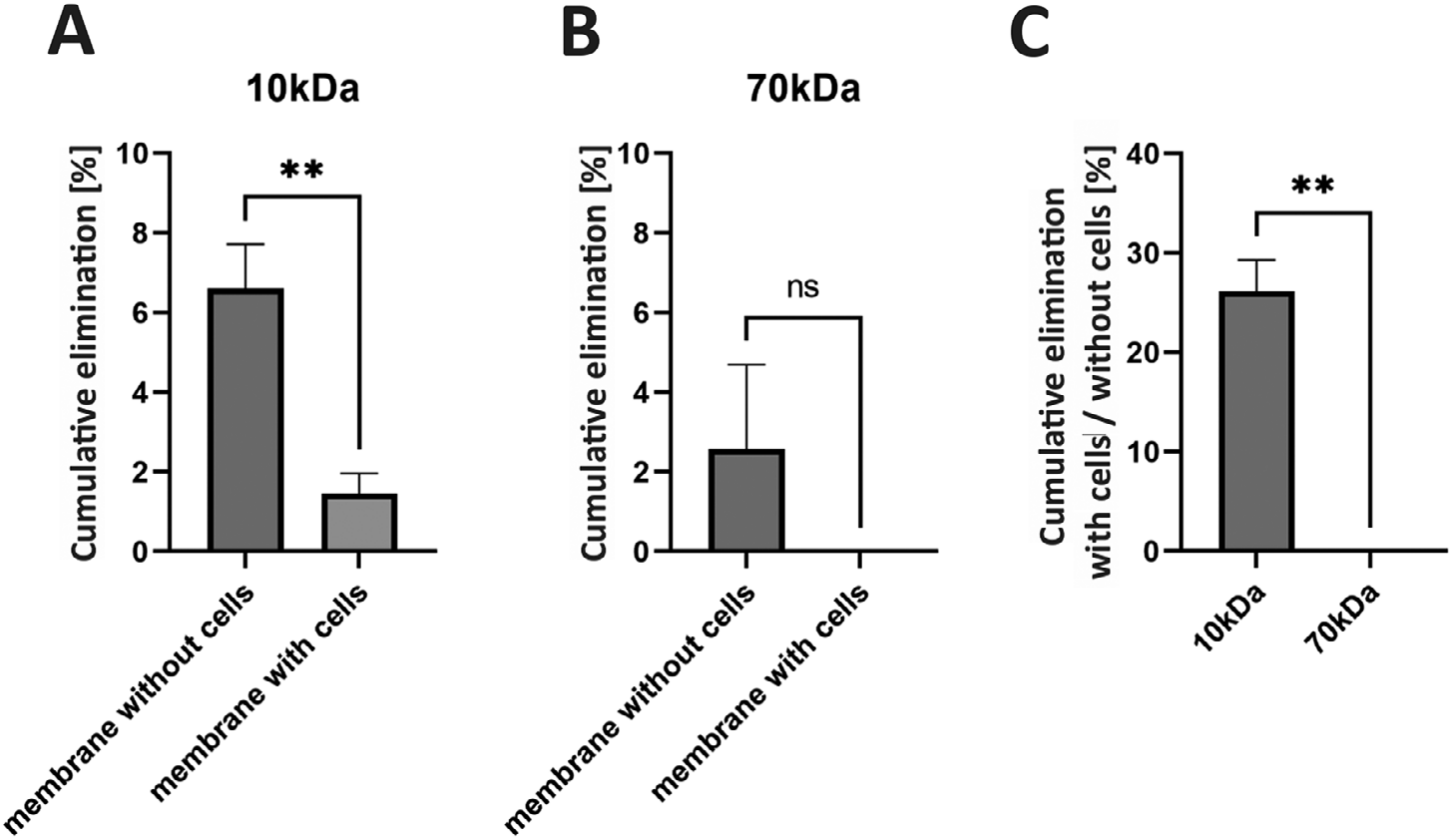
Permeability of the AFB. A) Cumulative elimination of 10 kDa dextran (added to the GEC‐side) through the AFB after 30 min.  ^**^ *p* < 0.01. B) Cumulative elimination of 70 kDa dextran (added to the GEC‐side) through the AFB after 30 min. C) Comparison of the cumulative elimination of 10kDa and 70kDa with podocytes and GECs on the membrane versus the membrane without cells. ^**^ *p* < 0.01. Unpaired *t*‐test, *n* = 3. Abbreviations: AFB: artificial filtration barrier, GECs: glomerular endothelial cells.

Different molecular weights of fluorescent dextrans were introduced to the endothelial site of the artificial GFB fixed between two rings under static conditions. Aligned fibers alone, without any cells, permitted the passage of both 10 and 70 kDa dextrans (**Figure** **5**A–C). After seeding glomerular endothelial cells and podocytes on opposite sides of the artificial GBM, 10 kDa dextran was still able to traverse the artificial GFB, though in lower amounts. After 30 min, ≈2% of the 10 kDa dextran added to the endothelial side was detectable on the podocyte side of the membrane (Figure 5A). In contrast, no 70 kDa dextran passed through the artificial GBM seeded with glomerular cells after 30 min, indicating its tightness (Figure 5B,C). Therefore, our in vitro GFB demonstrates permselectivity comparable to in vivo conditions.

### 2.6 Flow‐Induced Shear Stress Caused Cell Maturation with Fenestrae Development

**Figure 6:**
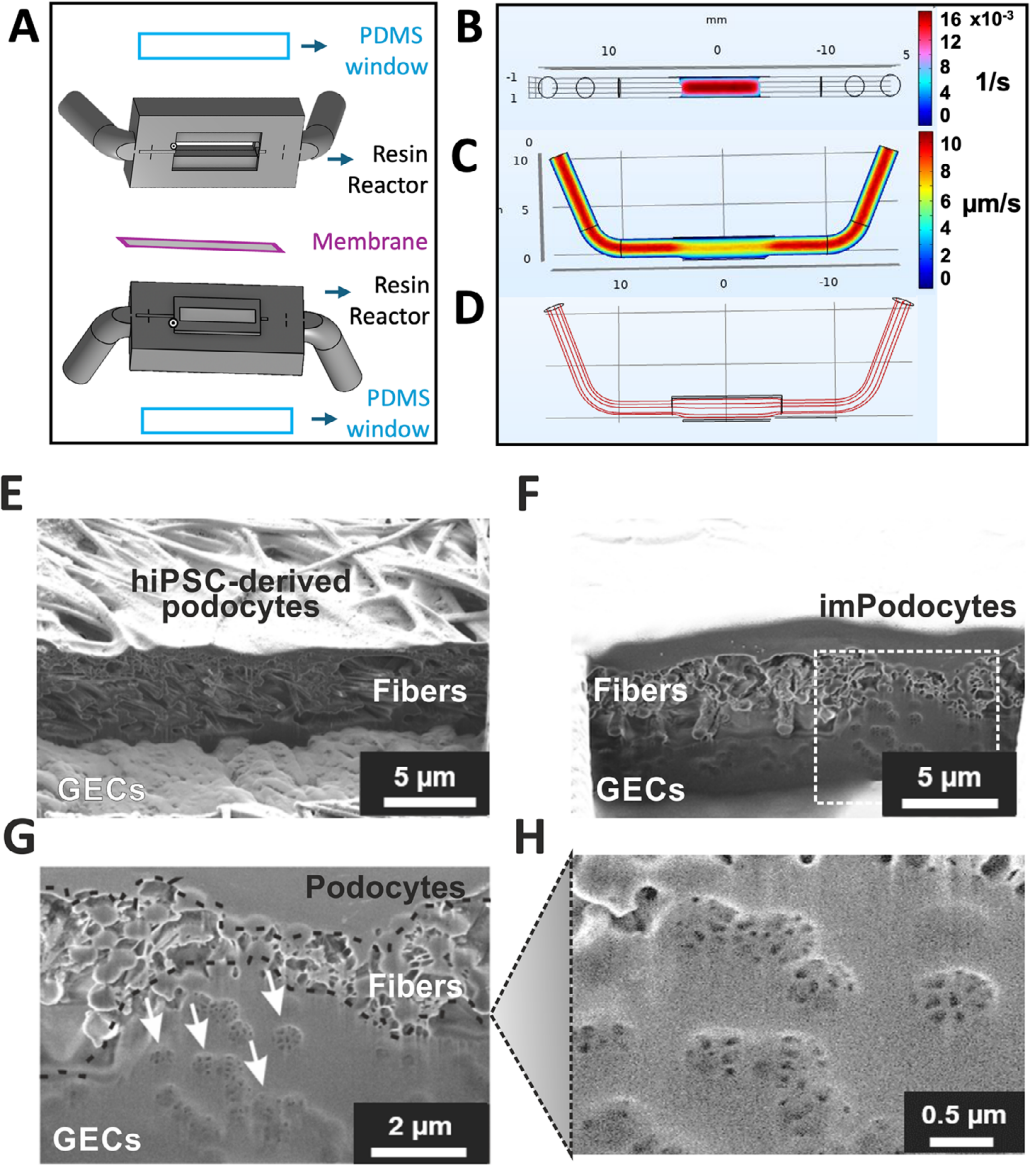
Design of the bioreactor and flow effects. A) Schematic of bioreactor components symmetrically composed of a PDMS window on a resin bioreactor in which the membrane is placed. B–D) COMSOL Multiphysics simulation of flow for 1 µL/min: shear rate (top), flow velocity (middle), laminar flow lines (bottom). E–H) SEM image of the cross‐section of the AFB membrane after three days under flow conditions, hiPSC‐derived podocytes (E) or immortalised podocytes (F) are on top, and GEC on the bottom with fibers in between. (G) Close up of the cross section highlighting fenestrae‐like structures on the GEC bottom side, (H) further magnification of the fenestrae‐like structures from (G‐arrows) for the imPodocytes and GECs, respectively, using SEM. Abbreviations: AFB: artificial filtration barrier. GEC: glomerular endothelial cells, hiPSC: human induced pluripotent stem cells, imPodocytes: human conditionally immortalized podocytes, SEM: Scanning electron microscopy. ​.

Next, we implemented flow into our model by incorporating the membrane in a bioreactor with the intention to improve endothelial cell maturation by shear stress. The bioreactor was designed on OnShape CAD software and printed with a PRUSA SLS1 Direct Light Processing (DLP) printer in Fotodent(R), a biocompatible resin (**Figure** **6**A). The advantages of these bioreactors over traditional organs‐on‐chip include the ability to design and print a stable, custom‐made device that can be sterilized and reused multiple times, while still achieving sub‐millimeter resolution with DLP Printers. Because the resin is not transparent and does not allow oxygen to pass through, a pre‐cast thin PDMS membrane was added to the reactor to facilitate oxygenation. The reactor is sealed mechanically with the membrane fixed between two parts that are compressed with pinchers. The bioreactor features two inlets and two outlets to enable parallel flow on both the endothelial and podocyte sides. A flow model in the bioreactor was simulated using the software “Comsol Multiphysics” to evaluate the velocities and shear stress of the laminar flow of the media acting on the cells on the membrane. This simulation aimed to optimize flow velocity and bioreactor design to better match physiological conditions (Figure 6B–D).

Glomerular endothelial cells were seeded first. The following day, podocytes were seeded on the opposite side of the membrane. Flow was introduced into the model using the bioreactor for 3 days (Figure S4B,C, Supporting Information). Due to the flow conditions, podocytes formed longer foot process‐like structures around the fibers (Figure 6E). Most importantly, the introduction of flow to our model prompted the development of fenestrae‐like structures in glomerular endothelial cells, serving as a critical indicator for improved maturation of those cells in co‐culture and under shear stress. This level of maturation of glomerular endothelial cell fenestrae has not been observed in other in vitro models previously (Figure 6F–H).

## 3 Discussion

The GFB consists of three layers. The first layer is built of specialized glomerular endothelial cells characterized by a high number of perforations called fenestrae. The second layer is composed of collagen IV, laminins, fibronectins, and proteoglycans organized in a GBM. The last layer is formed by podocytes with interdigitating foot processes that are bridged by slit diaphragms. All layers of the GFB are important for its specific permselectivity.^[^ ^30^ ^]^ There is an unmet need for in vitro GFB models to simulate physiological and pathophysiological conditions. 

Most existing in vitro models for the GFB used commercially available on‐chip devices,^[^ ^8^, ^9^, ^10^, ^11^, ^12^, ^13^, ^14^, ^15^, ^16^, ^17^ ^]^ tissue culture inserts or membranes^[^ ^10^, ^19^, ^20^, ^21^ ^]^ allowing to seed cells on opposite sides of a plastic membrane only under predefined conditions. For example, in the model by Li et al. 2016 commercially available and prescribed 1 µm porous membranes made of hydrophilic polytetrafluoroethylene (PTFE), polycarbonate or polyethylene terephthalate (PET) were used.^[^ ^10^, ^19^, ^20^, ^21^ ^]^ However, OOC systems rely on synthetic membranes, which are significantly thicker than human GBM and lack the necessary topographical features to support proper ECM remodeling and transmembrane crosstalk. Minimizing the membrane thickness enables realistic diffusion distances for molecules like albumin or creatinine. A correctly reconstructed thickness ensures realistic cell‐matrix interactions, signal transmission, and contributes to physiological pressure conditions and adhesion. Furthermore, most glomerular co‐culture models of the past used murine cell lines^[^ ^10^, ^12^, ^17^, ^19^ ^]^ or human immortalized podocytes^[^ ^11^, ^20^, ^31^, ^32^ ^]^ that do not resemble in vivo podocytes in terms of podocyte marker expression and foot processes.^[^ ^24^, ^25^ ^]^ Musah et al. established a differentiation protocol of hiPSCs to vascular endothelium and podocytes and co‐cultured them in a microfluidic kidney glomerulus chip for the first time.^[^ ^14^ ^]^ Although mechanical stimuli such as shear and tensile stresses are vital for the GFB's physiological functions,^[^ ^33^ ^]^ only a few previous models incorporated dynamic flow conditions.^[^ ^8^, ^34^, ^35^ ^]^ For example, Slater et al. used human cells in their GFB model but did not implement flow, thereby neglecting filtration aspects in their models.^[^ ^20^ ^]^ We used electrospinning to generate an artificial GBM which was subsequently seeded with human glomerular endothelial cells and human immortalized podocytes but also stem cell‐derived podocytes on opposite sides of this membrane. The surface of the nanofibrous membrane was either uncoated or functionalized with gelatin using polydopamine chemistry to improve cell adhesion, and fibers were characterized by electron microscopy, Raman spectroscopy, and FTIR spectroscopy. Different fiber orientations within the artificial membrane were tested, revealing that cell‐cell and cell‐material interactions, along with cell morphology, were optimally maintained when cells were co‐cultured on aligned fibers. To understand the role of surface modifications on cellular behavior, we examined the effects of polydopamine (PDA) and gelatin coatings on PLLA nanofibers. PDA deposition initially formed a conformal nanolayer, the self‐polymerization process resulted in nanoscale aggregate formation on the fiber surface. These aggregates, clearly visible in SEM images (Figure 1B), increased the local surface roughness of individual nanofibers.^[^ ^36^, ^37^, ^38^, ^39^ ^]^ Subsequent gelatin immobilization further enhanced roughness and introduced cell‐adhesive bioactive motifs such as Arginylglycylaspartic acid (RGD) sequences.^[^ ^40^, ^41^, ^42^, ^43^ ^]^ These changes improved cell attachment and spreading. Importantly, the coating‐induced roughness occurred independently of fiber arrangement, as both aligned and random mats underwent identical surface treatments. While surface roughness and biochemical functionalization primarily influenced cell adhesion, the underlying fiber orientation affected cellular morphology. Cells cultured on aligned fibers exhibited elongation along the fiber axis, whereas those on random fibers showed more isotropic spreading. In contrast, cells on uncoated pristine mats showed minimal adhesion and did not form confluent layers, regardless of fiber orientation, highlighting the essential contribution of surface chemistry to cell–material interactions. We observed that the amount of gelatin immobilized on aligned PLLA nanofiber mats was more than twice that on random mats. This significant difference cannot be attributed to a higher number of fibers, as both mat types were fabricated using the same amount of polymer solution. Instead, the increased gelatin attachment on aligned mats is explained by their unique structural characteristics. Aligned nanofibers form more open and linear networks with reduced fiber overlapping, thereby exposing a larger and more accessible surface area for surface modification and coating.^[^ ^42^, ^44^, ^45^, ^46^, ^47^, ^48^ ^]^ In contrast, randomly oriented mats create denser, interwoven structures that limit the diffusion and uniform adsorption of gelatin molecules due to shadowing effects and reduced surface exposure.^[^ ^45^ ^]^ Moreover, the aligned architecture facilitates more uniform polydopamine (PDA) deposition, which enhances the density of functional groups available for subsequent gelatin immobilization. The anisotropic surface of aligned fibers also supports better spreading of gelatin solution and more predictable protein adsorption.^[^ ^44^, ^49^ ^]^ Since gelatin contains bioactive motifs such as RGD sequences, which promote integrin‐mediated adhesion of podocytes and glomerular endothelial cells, the improved gelatin immobilization on aligned mats likely contributes to enhanced cell attachment.^[^ ^40^, ^41^, ^42^, ^43^ ^]^ To further support this explanation, SEM images of the glomerular co‐culture on uncoated aligned and random mats are provided in the supplementary materials, clearly highlighting the differences in cell morphology between these fiber orientations. This also aligns with previous findings showing that aligned fiber scaffolds enhance the differentiation of renal stem cells into podocytes.^[^ ^50^ ^]^ Thus, our surface‐functionalized artificial GBM supported a co‐culture of glomerular cells to mimic the GFB.  

Immortalized podocytes, used by others as well as in our model, have several limitations: Cells can undergo spontaneous dedifferentiation, and many podocyte‐specific markers are sparsely expressed or absent. This raises questions about the use of immortalized podocytes. In this study, all experiments involving podocytes were conducted under co‐culture conditions with glomerular endothelial cells across nanofiber membranes. This approach was based on our previous findings and consistent with earlier reports showing that co‐culture enhances podocyte survival, preserves morphology, and promotes the expression of key functional markers.^[^ ^51^ ^]^ Importantly, this design mimics the native glomerular filtration barrier, where podocytes and GECs reside on opposite sides of the glomerular basement membrane forming a physiologically relevant bilayered structure.^[^ ^52^, ^53^ ^]^ By employing electrospun nanofiber membranes as GBM analogs, we recreated this architecture in vitro. The observed uniform layer formation of podocytes on aligned fibers was therefore identified under these co‐culture conditions, which supported stable adhesion, spreading, and intercellular interactions across the membrane. Furthermore, not only immortalized podocytes survive on the artificial GBM, but also hiPSC‐derived podocytes, which revealed a morphology much more comparable to podocytes in vivo. HiPSC‐derived podocytes were generated using a protocol starting from fibroblasts obtained from a skin biopsy, followed by electroporation of stem cell plasmids and ultimately chemically defined differentiation into podocytes.^[^ ^26^ ^]^ Glomerular cells produced their own extracellular matrix and expressed cell‐type‐specific markers on the artificial GBM.

Additionally, we demonstrated that bidirectional cell‐cell communication between glomerular endothelial cells and podocytes is possible. This dual communication is crucial for the maturation and function of the GFB, as well as for maintaining glomerular integrity in vivo.^[^ ^27^, ^28^ ^]^

We evaluated the permeability of the artificial GFB by measuring the passage of dextrans of varying sizes through the barrier. Our results indicated that aligned fibers alone formed a significant barrier for 10 kDa and 70 kDa dextrans. After seeding glomerular endothelial cells and podocytes, the barrier became much tighter for 10 kDa dextran, which was still capable of passing through. However, the artificial GFB seeded with glomerular cells no longer permitted the passage of 70 kDa dextrans. In vivo, the functional cut‐off for the human GFB is also thought to be ≈70 kDa, and 10 kDa dextran injections in zebrafish showed cumulative elimination rates comparable to those in our in vitro model.^[^ ^54^ ^]^ Therefore, our artificial membrane demonstrated functionality very similar to in vivo conditions.

Shear stress that glomerular endothelial cells experience through blood flow and podocytes through urine flow within the Bowman capsule is important for cell maturation.  We established a low‐velocity flow on both sides of our model by integrating the membrane into a bioreactor. The advantages of these bioreactors over traditional organs‐on‐chip include the ability to design and print a stable, custom‐made device that can be sterilized and reused multiple times, while still achieving sub‐millimeter resolution with DLP Printers. Although physiological stresses are higher than the ones achieved in this study these rates were chosen to approximate the shear stress experienced by GECs in vivo and utilized in prior microfluidic studies modeling glomerular or vascular interfaces, while maintaining a lower perfusion rate on the podocyte side to avoid shear‐induced dedifferentiation, which podocytes are known to be sensitive to.

We demonstrated the flow‐dependent formation of glomerular endothelial cell fenestrae‐like structures that did not develop in other in vitro glomerular endothelial cell models. The podocyte foot process‐like structures also matured following exposure to shear stress.

In summary, we have shown that our artificial GFB facilitates in vivo‐like transmembrane intercellular crosstalk, ECM production, cell maturation, and perm‐selectivity. Compared to other 3D models of the GFB published prior to ours, our model presents several advantages and innovations: First, our artificial GBM offers design flexibility in terms of tunable porosity, control over fiber diameter, fiber orientation, and surface modification, allowing the exploration of the effects of surface topography on a GFB in‐vitro model. Previously published models typically use commercially available chips that are not customized or just random fibers. Second, our surface modification methods enable the functionalization of the artificial GBM with bioactive cues for improved cell adhesion and biomimicry. We employed gelatin coating for membrane biofunctionalization, which offers a cost‐effective and biologically relevant alternative to more expensive ECM proteins like laminin‐511, used in previous models.^[^ ^40^, ^41^, ^42^, ^43^ ^]^ Gelatin, being rich in RGD sequences, supports integrin‐mediated adhesion and simulates key aspects of the glomerular basement membrane. Despite its simplicity and affordability, we observed comparable outcomes in cell attachment, viability, and morphological maturation, suggesting its suitability for scalable and translational kidney models.

We possess state‐of‐the‐art instruments to characterize and validate the in vitro models, allowing us to gather critical information about the model's suitability and the potential to adjust it further based on cell responses. We provide ultrastructural evidence of endothelial fenestration using scanning electron microscopy (SEM) and focused ion beam (FIB), offering a high‐resolution view of structural maturation that, to our knowledge, surpasses what has been previously reported in vitro. Third, our functional assays, which include measuring permeability for dextrans of varying sizes as an indicator of the filtration barrier's leakiness and assessing paracrine communication among the GFB cells, make the model appropriate for future applications involving in vitro studies of pathophysiological conditions and drug testing.

## 4 Experimental Section

### Generation of Electrospun Membranes and Their Surface Modification

A nanofiber mesh was fabricated using electrospinning to artificially mimic the glomerular basement membrane. A 2% solution of poly‐L‐lactic acid (PLLA) (Purasorb® PL65, Corbion, Amsterdam, Netherlands) was prepared in HexaFluoroIsoPropanol (HFIP) (Sigma–Aldrich, Germany). The prepared solution was then extruded at a rate of 1 mL h⁻¹ using a syringe pump (World Precision Instruments, Sarasota, Florida, USA) through a 27G nozzle, with a voltage of 13 kV applied to the nozzle. The nanofibers were collected on aluminum foil covering a rotating grounded mandrel (Ø 10 cm), positioned 15 cm from the nozzle. Randomly oriented nanofibers were collected on the mandrel, which rotated at 180 rpm. To achieve aligned nanofibers, the collector setup was modified by replacing the aluminum foil with a Teflon® film decorated with copper microwires spaced 1 cm apart and wrapped around the grounded mandrel, which rotated at 500 rpm to collect the aligned nanofibers.

For surface coating with polydopamine, the membranes were dip‐coated in a dopamine hydrochloride (2 mg mL⁻¹, Sigma–Aldrich, Germany) solution in Tris‐HCl buffer (10 mmol, pH 8.5) under mild shaking for 4 h, as previously described.^[^ ^42^ ^]^ Subsequently, the polydopamine‐coated nanofibrous membranes were immersed in a gelatin type B (3 mg mL⁻¹, Sigma–Aldrich, Germany) solution in Tris‐HCl buffer (10 mmol, pH 8.5, overnight while shaking. The gelatin‐immobilized membranes were then washed several times to remove residual gelatin from the fibrous mesh and dried at room temperature.

### Evaluation of Polydopamine Coating and Gelatin Immobilization

We conducted the microBCA assay (Thermo Fisher Scientific) to quantify the polydopamine coating on the nanofibers, following the previously reported method.^[^ ^47^ ^]^ Membrane samples were cut into 0.5 cm^2^ squares and incubated with microBCA working solution (200 µl) for 2 h at 37 °C. The reacted working solution was then measured by absorption at 562 nm using a Tecan Spark® Multimode Microplate Reader (Tecan Trading AG, Switzerland) to determine the coating amount. Concurrently, standards were recorded, and the values were plotted against a standard curve for accurate determination of the polydopamine coating amount. Likewise, the microBCA assay was employed to indirectly quantify gelatin immobilization by measuring the remaining gelatin in the supernatant.^[^ ^55^ ^]^ Gelatin release from the membrane was measured over 1, 3, 6, 9, 12, and 24 h by keeping the membranes in PBS at 37°C while gently shaking; supernatant (50 µL) was collected and evaluated as previously described. The surface chemistry of the membranes, including PLLA Nanofibers (PL‐NFs), PLLA Dopamine‐coated Nanofibers (PLDA‐NFs), and PLLA Gelatin‐modified Nanofibers (PLGel‐NFs), was analyzed using Raman spectroscopy. Membrane samples (1 cm^2^) were placed under a DXR Raman Microscope (Thermo Scientific, USA), equipped with a 10x objective. The membranes were irradiated with a 780 nm laser at an intensity of 15 kW. Raman spectra were recorded and processed using Omnia software to identify chemical composition and surface modifications.

### Surface Characterization of Nanofibers

Scanning electron microscopy (SEM) was conducted on the nanofiber membranes to visualize their surface morphology through secondary electron images. The samples were sputter‐coated with a 4 nm platinum layer using a Leica EM ACE600 sputter coater (Leica Microsystems GmbH, Wetzlar, Germany) and examined with a Zeiss Crossbeam 340 Field‐Emission Electron Microscope (Zeiss Microscopy, Oberkochen, Germany) at an acceleration voltage of 2 kV. To further assess the 3D structure and membrane thickness, Gallium Focused Ion Beam (Ga‐FIB) imaging was employed. To evaluate the membrane topography and fiber morphology, SEM images were analyzed with Image J. The porosity of the electrospun mat was calculated from the ratio between membrane mass and bulk PLLA, as previously shown.^[^ ^22^ ^]^ Additionally, atomic force microscopy (AFM) was performed under ambient conditions using a Bruker Multimode 8 SPM system in tapping mode. Silicon cantilevers (OMCL‐AC160TS, Olympus) with a resonance frequency of ≈300 kHz and a spring constant of ≈26 Nm⁻¹ were used. Carbon adhesive pads were employed to secure the samples, including gelatin, PLLA, and PDA‐coated membranes, onto magnetic metal discs for surface roughness and topography analysis.

### Design and Fabrication of a 3D‐Printed Bioreactor for Bilayer Culture

To support bilayer culture under static conditions, we designed insert rings that facilitate the culture process, as described earlier^56^. These rings were 3D printed using Fotodent biocompatible resin (FotoDent guide 385 and 405 nm, Dreve, Germany) with a DLP PRUSA SL1S speed printer. The membranes are securely fixed between two 3D‐printed rings. For the bioreactor, a millifluidic design was developed using Computer‐Aided Design (CAD) software, OnShape. The bioreactor features a central channel measuring 2 mm in width, 1 mm in height, and 10 mm in length, with side wings designed for tubing connections. The top of the channel is open to allow for the insertion of a Polydimethylsiloxane (PDMS) membrane, which facilitates oxygenation. This system is also 3D‐printed using the DLP Prusa printer (Prusa SLS1 speed, Joseph Prusa Research, Czech Republic) with Fotodent resin at a layer height of 0.025 mm. Sylgard PDMS is prepared according to the manufacturer's specifications, cast to form a 0.5 mm thick membrane, and subsequently cured. The PDMS membrane serves as the bioreactor's window and is sealed using a biocompatible silicone (Dublisil, Dreve Dentamid GmbH, Unna, Germany) to ensure watertightness. After the membrane is securely placed between the two halves of the bioreactor, it is tightened using screw‐driven pinchers (A. Hartstein, Germany) and connected to perfusion tubing via Luer lock. Two perfusion pumps (Dissolution Accessories, Oosterhout, Netherlands) are equipped with syringes filled with media, connected to opposite sides of the bioreactor, and programmed for perfusion. To verify laminar flow and shear stress on the membrane, we utilized the COMSOL Multiphysics software for fluid dynamic assessment for laminar flow by setting the walls, inlet, and outlet as boundary conditions to match experimental values.

### Cell Culture of Human Immortalized Podocytes and Glomerular Endothelial Cells

A conditionally immortalized human podocyte cell line has been developed by Moin Saleem, Children's and Renal Unit and Bristol Renal, University of Bristol, by retroviral transfection of a nephrectomy specimen with the temperature‐sensitive *SV40‐T* gene.^[^ ^57^ ^]^ Therefore, cells can be expanded under permissive conditions at 33 °C. When cultivated at 37 °C, the transgene becomes inactivated, cells enter a growth arrest, and undergo terminal differentiation. Human podocytes were cultured in RPMI Medium 1640 (Gibco, ThermoFisher Scientific, Waltham, MA, USA), supplemented with heat‐inactivated fetal bovine serum (10%, FBS, PAN‐Biotech, Aidenbach, Germany), penicillin‐streptomycin (1%, Sigma–Aldrich, Merck, St. Louis, MO, USA), and insulin‐transferrin‐selenium (0.1%, ThermoFisher Scientific, Waltham, MA, USA). Primary human glomerular microvascular endothelial cells (glomerular endothelial cells ACBRI 128) were purchased from Cell Systems, Kirkland, WA, USA, and maintained in commercially available endothelial cell media (VascuLife VEGF‐Mv Medium, LifeLine Cell Technology, ThermoFisher Scientific, Waltham, MA, USA) containing rhFGF basic (5 ng mL⁻¹, ascorbic acid (50 µg mL⁻¹), hydrocortisone hemisuccinate (1 µg mL⁻¹), L‐glutamine(10 mmol), rhIGF‐1 (15 ng mL⁻¹), rhEGF (5 ng mL⁻¹), rhVEGF (5 ng mL⁻¹), heparin sulfate (0.75 U mL⁻¹), fetal bovine serum (5%), gentamycin (30 mg mL⁻¹), and amphotericin B (15 µg mL⁻¹) (all supplements, LifeLine® Cell Technology, Thermo Fisher Scientific, Waltham, MA, USA).

### HiPSC‐Culture and Differentiation

HiPSC lines were produced by Rose et al.^[^ ^26^ ^]^ As previously described, human material was obtained from skin biopsies of healthy volunteers who provided written informed consent. Briefly, dermal fibroblasts cultivated from skin biopsies were episomally reprogrammed with a plasmid mix (pCXLE‐hOCT3/4, pCXLE‐hSK, pCXLE hMLN) using electroporation. For expansion and maintenance, hiPSCs were cultured on a Matrigel‐coated (Corning, 354277, at a final concentration of 2.5 µg mL⁻¹) Nunc™ 6‐well plate with mTeSR1 (Stem Cell Technologies, 85850) culture medium supplemented with Pen/Strep (1%, Sigma–Aldrich, P4333). The medium was replaced every two days. Cells were passaged once or twice a week using Accutase (Gibco, A1110501). To enhance cell survival, both the Accutase and the cell medium were supplemented with Rho‐associated kinase (10 µmol, Y‐27623, Tocris, 1254) inhibitor for 24 h.

HiPS‐derived podocytes were generated using previously described chemically defined growth medium conditions.^[^ ^14^, ^16^, ^26^ ^]^ The differentiation protocol is summarized in Figure 3A. First, cells were seeded at a high density (1 × 10⁴ hiPSC cm^−^^2^) onto Nunc™ EasYFlask™ T75 cell culture flasks coated with Matrigel as described above. After overnight attachment, the prewarmed human mesoderm differentiation medium (DMEM/F12 (GibcoTM, 11320074) supplemented with B27 supplement (1x, GibcoTM, 17504044), Pen/Strep (1%), CHIRR99021 (3 µmol, Sigma–Aldrich, 252917‐06‐9), Activin A (50 ng mL⁻¹ Stem Cell Technologies, 78001.1), and Y27632 (10 µmol) was added to the cell culture. After two days, it was replaced with the intermediate mesoderm differentiation medium (same as the mesoderm differentiation medium except for the absence of Y27632 and with an additional BMP7 (50 ng mL⁻¹, Peprotech, 120‐03P)). The medium was changed every two days for the next 14 days. For terminal differentiation into hiPSC‐derived podocytes, cells were cultured in the podocyte end‐differentiation medium (intermediate mesoderm differentiation medium with the addition of VEGF (25 ng mL⁻¹, Thermo Scientific, PHC9394) and All‐trans retinoic acid (0.5 µmol, Stem Cell Technologies, 72262)). After differentiation, hiPSC‐derived podocytes were maintained in the podocyte cell medium as described above.

### Ethical Statement

All preparations received approval from the local ethics committee (Approval no. 251_18B, Uniklinikum Erlangen). All experiments were conducted in accordance with the guidelines and regulations of the ethics committee at Uniklinikum Erlangen. It confirms that informed consent was obtained from all participants.

### Artificial GFB Characterization in Static Culture Conditions

For artificial GFB under static conditions, PLLA membranes were secured between two supporting rings and sterilized with a UV lamp. A suspension of 200,000 glomerular endothelial cells was seeded into the inner portion of the supporting rings (radius 3 mm) onto the membrane (7 × 10^5^ cm^−^^2^). The prewarmed human endothelial medium was added outside the supporting rings into the well, filling it to the top of the supporting rings. The following day, the assembly was inverted. A total of 7 × 10^5^ cm^−^^2^ pre‐differentiated human imPodocytes or 3.5 × 10^5^ cm^−^^2^ hiPSC‐derived podocytes were seeded onto the membrane. The well was filled with a mixture of human endothelial and podocyte medium to the brim of the supporting rings. The next day, the constructs were placed into a 6‐well plate containing 8 mL of mixed medium. On day four, the artificial GFB was characterized using the methods outlined below.

### Artificial GFB Characterization in Dynamic Culture Conditions with the Bioreactor

After sterilization with ethanol and/or UV light, a membrane was affixed between two parts of the bioreactor. A cell suspension containing 1 × 10^6^ cm^−^^2^ glomerular endothelial cells was seeded into one chamber. The next day, glomerular endothelial cells were provided with fresh medium, and a cell suspension of 1 × 10^6^ cm^−^^2^ imPodocytes or 0.5 × 10^6^ cm^−^^2^ hiPSC‐derived podocytes was added to the second chamber. On the following day, the bioreactor was connected to syringe pumps via tubing. Flow rates of 0.5 µL min^−1^ for podocytes, 1 µL min^−1^ for glomerular endothelial cells were maintained for three days.

### Live Dead Assay

Cell viability was assessed using the LIVE/DEAD™ Cell Imaging Kit (ThermoFisher Scientific, Waltham, MA, USA). The kit was utilized according to the manufacturer's instructions. Briefly, two components were mixed and added to the samples (1:1 with cell medium). After a 15‐min incubation, fluorescence was visualized using the EVOS™ Imaging System (ThermoFisher Scientific, Waltham, MA, USA).

### Scanning Electron Microscopy Analysis of Artificial GFB

The cells grown on the membrane, referred to as GFB samples, were fixed using a solution of paraformaldehyde (4.5%, PFA) and glutaraldehyde (2%) in HEPES buffer (0.1 mol, pH 7.4). The samples were then dehydrated through a graded ethanol series and dried overnight with hexamethyldisilazane (HMDS). Following this preparation, the samples were sputter‐coated, and scanning electron microscopy (SEM) was performed as previously described (Chapter 4.3).

### Immunofluorescent Staining

Samples were washed with 1× PBS and fixed with PFA (4%, Roth, Karlsruhe, Germany) for 15 min. After rinsing, samples were incubated with a blocking solution (1× PBS containing normal goat serum (10%, NGS, Abcam, Cambridge, UK), bovine serum albumin (1%, BSA, Karlsruhe, Germany), and Triton X–100 (0.5%, Merck, Darmstadt, Germany)) for 1 h at room temperature (RT). Subsequently, samples were incubated with primary antibodies (see Table S1, Supporting Information) diluted in 1× PBS containing NGS (3%) and BSA (1%) overnight at 4 °C. The following day, samples were washed three times with 1× PBS and labeled with fluorescent secondary antibodies (goat anti‐rabbit 647 (A‐31634, Thermo Fisher Scientific, Waltham, MA, USA), goat anti‐mouse 488 (A‐21244, Thermo Fisher Scientific, Waltham, MA, USA) in the dark at RT for 1 h. To label the actin cytoskeleton, Alexa Fluor 555 Phalloidin (A‐34055, Thermo Fisher Scientific, Waltham, MA, USA) was applied along with the secondary antibodies.

Next, nuclei were stained with DAPI (Thermo Fisher Scientific, Waltham, MA, USA; 1:200 dilution in 1× PBS for 15 min in the dark). After washing with 1× PBS, samples were mounted with a drop of Fluoromount‐G™ Mounting Medium (Invitrogen, Thermo Fisher Scientific, Waltham, MA, USA) between a cover slip and a microscope glass slide.

### VEGF‐A Electroporation

A human VEGFA sequence coupled with a GFP sequence was cloned into a mammalian gene expression vector (pLV[Exp]‐Puro‐CMV>3xFLAG/ORF_Stuffer, #VB900138‐3673 twt, Vectorbuilder, Chicago, IL, USA). After transformation and expansion in E. coli, the plasmid was purified using the QIAprep Spin Miniprep Kit (QIAGEN). To transfect the VEGFA‐GFP reporter plasmid, terminally differentiated hiPSC‐derived podocytes were prepared by washing with PBS and Opti‐MEM Medium (ThermoFisher Scientific). Then, plasmid (5 µg per 1–2.5 million cells) was electroporated at 280 V, Discharge Interval 50, 151 MSEC (Progenetor 11, Hoefer). Cells were seeded onto the aligned membrane colonized by glomerular endothelial cells from the opposite side the day before. After three days of co‐culturing, the artificial GFBs were fixed and mounted as described above and visualized using confocal microscopy.

### MiRNA Transfection

After seeding and attachment on the aligned membrane, glomerular endothelial cells were transfected with 200 nmol Alexa555‐tagged miR‐192 (MirVanaTM, custom miRNA mimic, Thermo‐Fisher Scientific) for 4 h using Lipofectamine 2000 and Opti‐MEM Medium (Thermo Fisher Scientific) following the manufacturer's protocol. The following day, terminally differentiated hiPSC‐derived podocytes were labeled with eBioscience Cell Proliferation Dye eFluor 450 (Invitrogen, at a final concentration of 2 µM) in accordance with the manufacturer's instructions and seeded on the opposite side of the membrane. After 24 h of co‐culturing, the artificial GFBs were fixed, nuclei were stained with SYTO Deep Red (Thermo‐Fisher Scientific) for 5 min, rinsed, mounted as previously described, and visualized using confocal microscopy.

### Permeability Assay

Artificial GFBs, either without cells or seeded with glomerular endothelial cells and imPodocytes, were transferred to a clean well. A dextran mixture containing Cascade Blue‐labeled 10 kDa and Texas Red‐labeled 70 kDa dextrans (Thermo‐Fisher Scientific), diluted in a human endothelial and podocyte medium mix (100 µg mL^−1^, 50 µL), was added to the inner section of the supporting rings (radius 2 mm). Pure human endothelial and podocyte medium mix was then added outside the supporting rings into the well. After 30 min of incubation at 37 °C, the artificial GFB construct was removed, and the remaining liquid was collected. Fluorescence was measured using the GloMax‐Multi+Detection System (Promega) at 405/495 and 625/660 excitation/emission wavelengths, respectively.

### Statistical Analysis

Data is reported as mean ± standard deviation, with a minimum of *n* = 3 replicates per condition unless stated otherwise. Statistical analysis was conducted using GraphPad Prism (version 9.5.1) or MATLAB® (version R2022a) employing a two‐tailed unpaired t‐test. Statistically significant differences between groups are denoted by ^*^(*p* < 0.05) or ^**^(*p* < 0.01).

## Conflict of Interest

The authors declare no conflict of interest.

## Author Contributions

C.M. and A.R. contributed equally to this work. C.M. contributed to conceptualization, data collection, data analysis, methodology, visualization, and writing. A.R. was involved in conceptualization, data collection, data analysis, methodology, visualization, and writing. P.S. performed SEM imaging, while V.S. and F.W. carried out AFM measurements. J.G. was responsible for funding acquisition and supervision. M.S. contributed to writing the review. T.A. and J.M.D. share correspondence responsibilities and contributed equally to project planning, administration, supervision, funding acquisition, original draft preparation, and writing review and editing.

## Supporting information



Supporting Information

## Data Availability

The data that support the findings of this study are available from the corresponding author upon reasonable request.
