# Supplementary material for: Biofabrication of a Filtration Barrier by Integrating Electrospun Membranes and Flow in a Glomerular Co‐Culture
Source: Adv Healthc Mater. 2025 Jul 3;14(23):2501235. doi: 10.1002/adhm.202501235 (PMC12417763; doi:10.1002/adhm.202501235)
Supplement: Supplementary file 1 — Supporting Information [file ADHM-14-0-s001.docx]

Supporting Information

**Biofabrication of a filtration barrier by integrating electrospun membranes and flow in a glomerular co-culture**

*Camilla Mussoni †, Anna Rederer †, Vladimir Stepanenko, Frank Würthner, Philipp Stahlhut, Jürgen Groll, Mario Schiffer, Taufiq Ahmad ^*^, Janina Müller-Deile ^*^*

***Table S1: Primary antibodies***

| **Antigen (Clone)** | **Host** | **Company, Cat. No.** | **Dilution** |
| --- | --- | --- | --- |
| Collagen IV | rabbit | Ab6586, Abcam, Cambridge, UK | 1: 200 |
| Laminin 5 | mouse | Ab77175, Abcam, Cambridge, UK | 1: 200 |
| CD31 | mouse | Ab9498, Abcam, Cambridge, UK | 1: 200 |
| Synaptopodin | rabbit | 21064-1-AP, Proteintech, Rosemont, IL, USA | 1: 200 |
| VEGF (VG1) | mouse | MA1-16629, Invitrogen, Carlsbad, USA | 1: 200 |


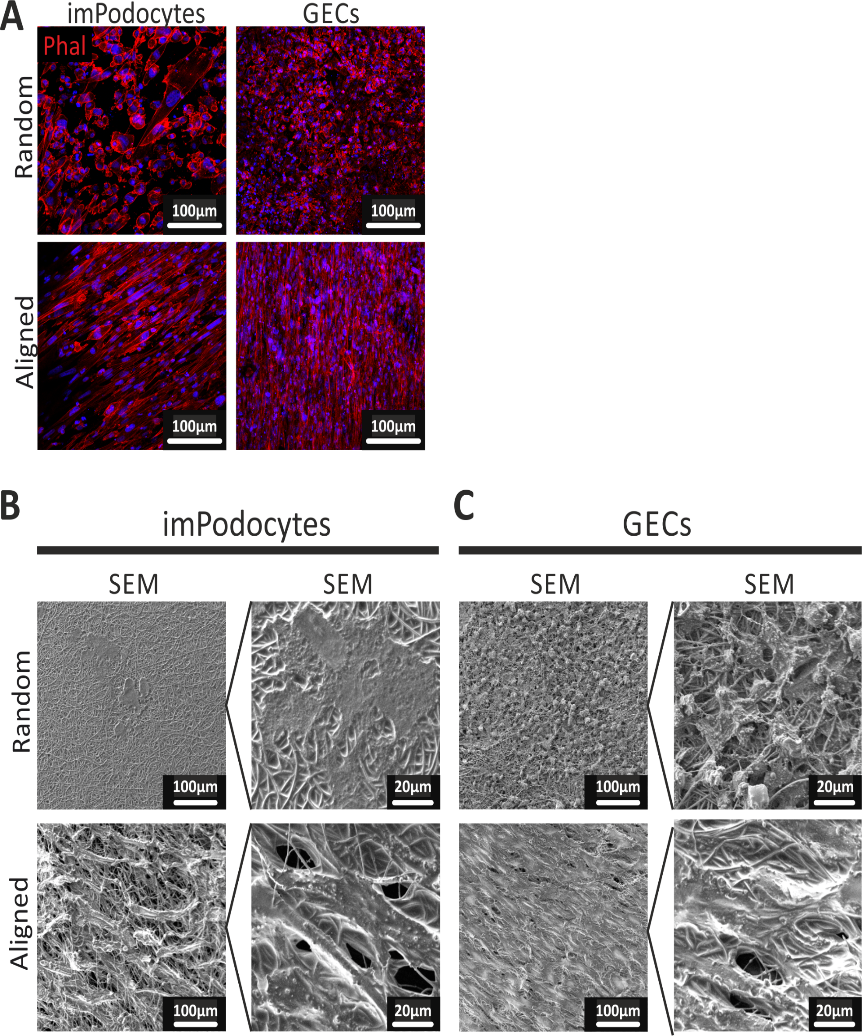


**Figure S1: Co-culture of human conditionally immortalized podocytes (imPodocytes) and glomerular endothelial cells (GECs) on the electrospun non-coated PLLA-membrane.**Cell morphology and layer integrity of imPodocytes (left panels) and GECs (right panels) on the membrane with random (upper panels) and aligned (bottom panels) topography visualized using confocal microscopy with phalloidin (Phal) staining (A) and SEM (B-C). Scale bar 100 µm and 20 µm.​Abbreviations: GECs: glomerular endothelial cells, ImPodocytes: human conditionally immortalized podocytes, SEM: Scanning electron microscopy.


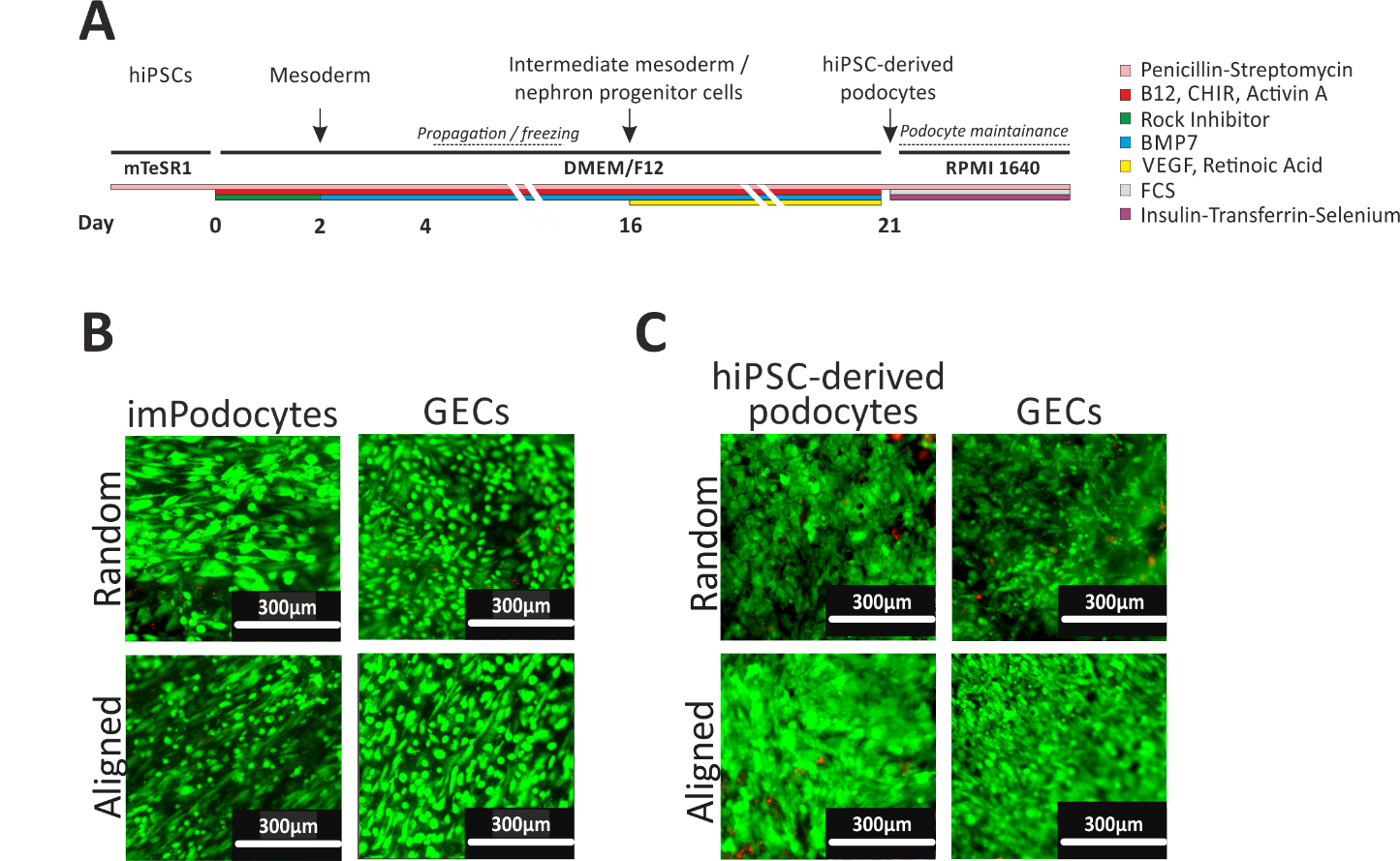


**Figure S2: Cell differentiation and viability*.*** (A) Schematic timeline and medium composition for hiPSCs-differentiation into podocytes. ​ (B-C) Live/dead staining of imPodocytes (B) or hiPSC-derived podocytes (C) and GECs on the membrane with random (upper panels) and aligned (bottom panels) topography visualized using EVOS microscope.

Abbreviations: GEC: glomerular endothelial cells, hiPSC: human induced pluripotent stem cells, imPodocytes: human conditionally immortalized podocytes


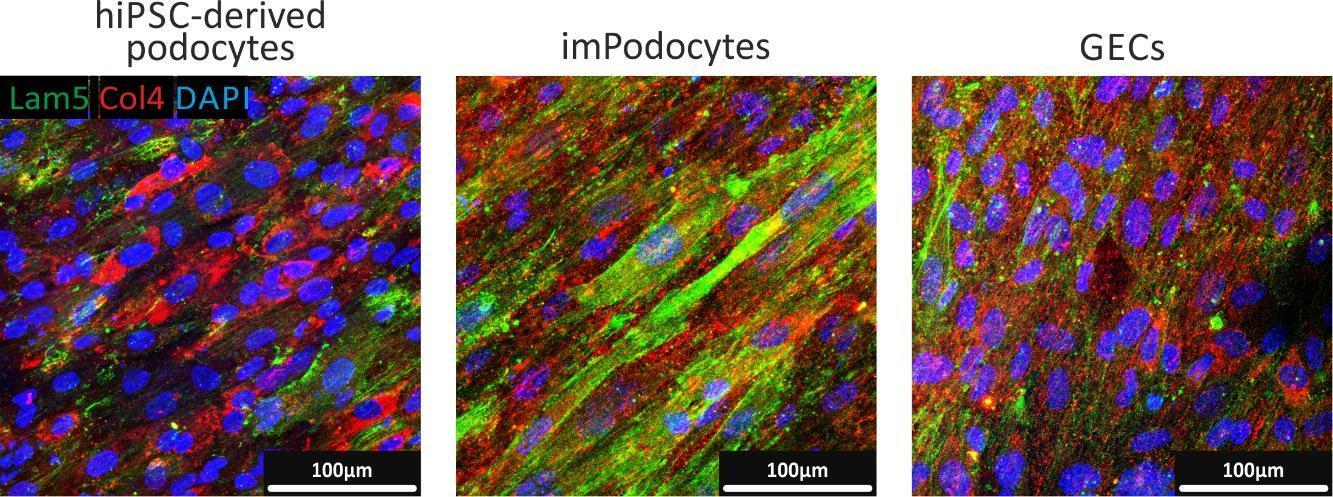


**Figure S3: ECM production of cells co-cultured on the aligned membrane.** Immunofluorescent staining for laminin 5 (Lam5; green), collagen IV (Col4; red), and DAPI nuclei staining (blue) of hiPSC-derived podocytes (left panel), imPodocytes (middle panel) and GECs (right panel) cultured on the electrospun polydopamine- and gelatin-coated PLLA-membrane with aligned topography visualized using confocal microscopy. Scale bar 100 µm. ​Abbreviations: ECM: extracellular matrix, GEC: glomerular endothelial cells, hiPSC: human induced pluripotent stem cells, imPodocytes: human conditionally immortalized podocytes

**
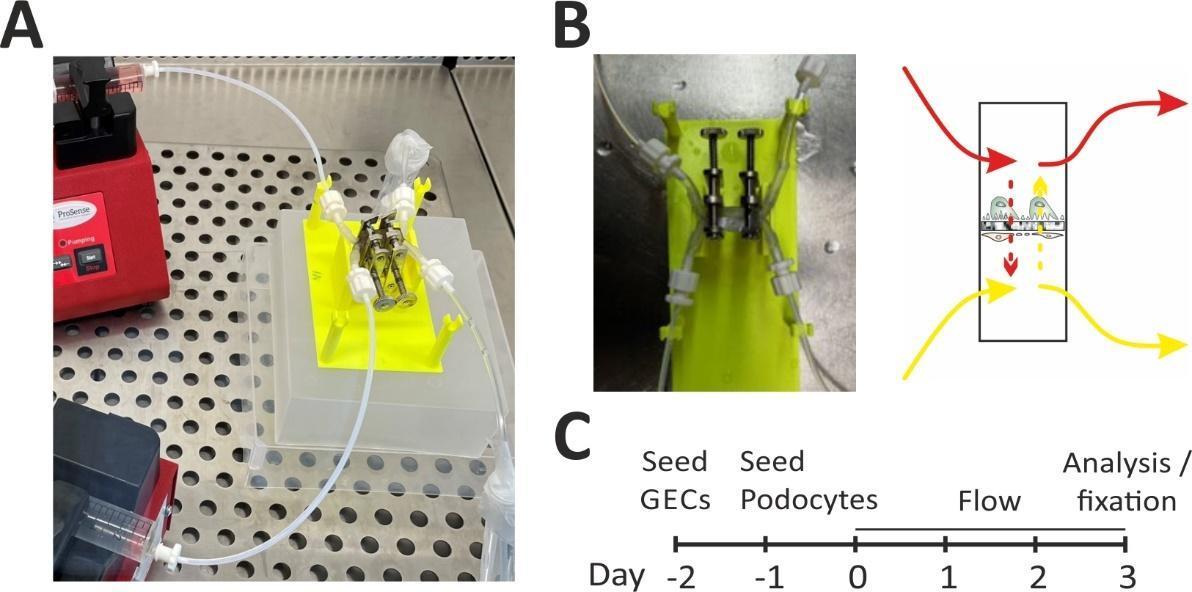
**


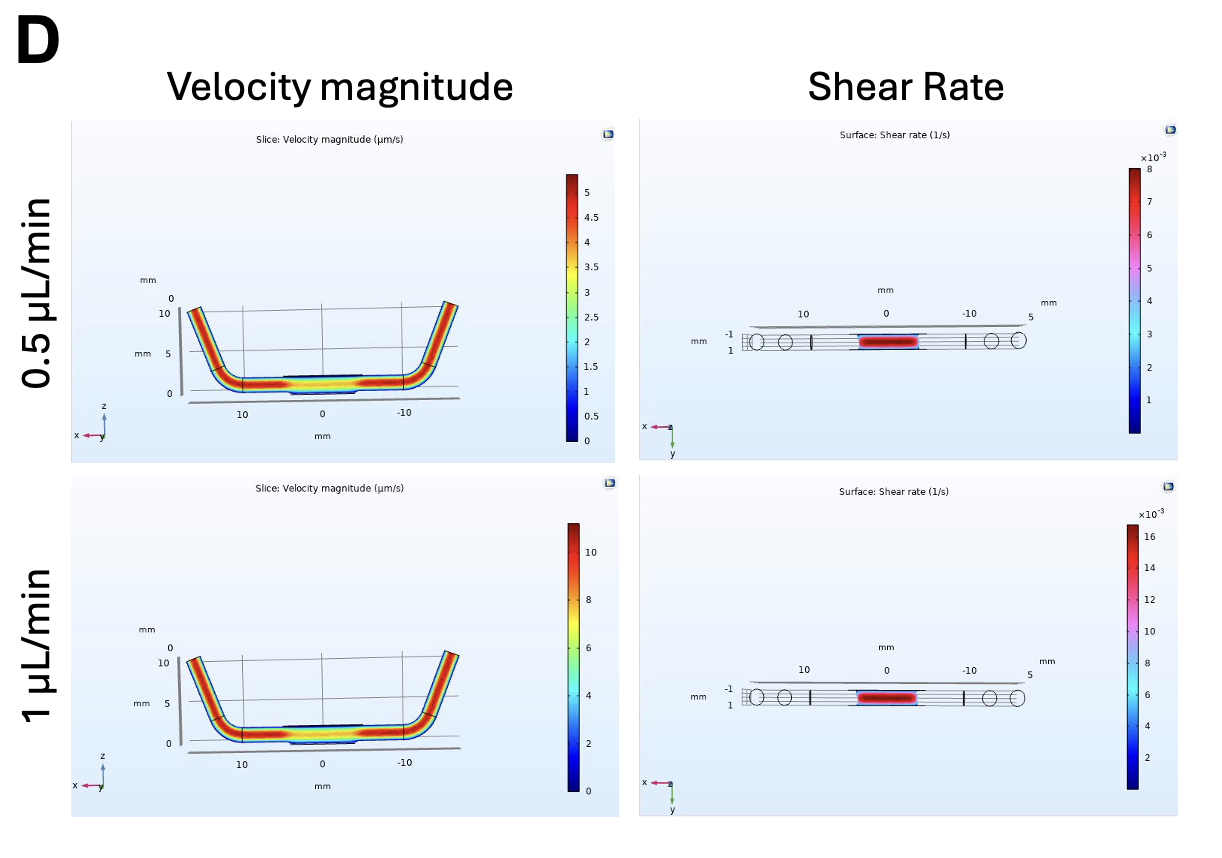


**Figure S4: Bioreactor perfusion setup and experimental timeline.** (A) Complete bioreactor perfusion setup. (B) Bioreactor with tube connections (left) and schematic directions of flow (right). (C) Experimental timeline. (D) Comsol multiphysics simulation results for velocity magnitude and sheat rate for 0.5 and 1 µL min^-1^. Abbreviations: GEC: glomerular endothelial cells, Podocytes: human conditionally immortalized podocytes
